# Different Teams, Same Conclusions? A Systematic Review of Existing Clinical Guidelines for the Assessment and Treatment of Tinnitus in Adults

**DOI:** 10.3389/fpsyg.2017.00206

**Published:** 2017-02-22

**Authors:** Thomas E. Fuller, Haula F. Haider, Dimitris Kikidis, Alec Lapira, Birgit Mazurek, Arnaud Norena, Sarah Rabau, Rachelle Lardinois, Christopher R. Cederroth, Niklas K. Edvall, Petra G. Brueggemann, Susanne N. Rosing, Anestis Kapandais, Dorte Lungaard, Derek J. Hoare, Rilana F. F Cima

**Affiliations:** ^1^Clinical Psychological Science, Faculty of Psychology and Neuroscience, Maastricht UniversityMaastricht, Netherlands; ^2^Adelante, Centre of Expertise in Rehabilitation and AudiologyHoensbroek, Netherlands; ^3^ENT Department of Hospital, Cuf Infante Santo - Nova Medical SchoolLisbon, Portugal; ^4^Department of Otorhinolaryngology, Head and Neck Surgery, National and Kapodistrian University of Athens, Hippocrateion General HospitalAthens, Greece; ^5^ENT Specialist, Institute of Health Care, Mater Dei HospitalMalta, Malta; ^6^Tinnitus Center, Charite University HospitalBerlin, Germany; ^7^Laboratory of Adaptive and Integrative Neuroscience, Centre National de la Recherche Scientifique, Fédération de Recherche, Aix-Marseille UniversitéMarseille, France; ^8^Faculty of Medicine and Health Sciences, Campus Drie Eiken, University of AntwerpAntwerp, Belgium; ^9^Department of Physiology and Pharmacology, Karolinska InstitutetStockholm, Sweden; ^10^Department of Clinical Research, Faculty of Health Sciences, University of Southern DenmarkOdense, Denmark; ^11^Department of Nordic Studies and Linguistics, Copenhagen UniversityDenmark; ^12^NIHR Nottingham Hearing Biomedical Research Unit, Division of Clinical Neuroscience, School of Medicine, University of NottinghamNottingham, UK

**Keywords:** tinnitus, clinical guidelines, assessment, treatment, systematic review

## Abstract

**Background:** Though clinical guidelines for assessment and treatment of chronic subjective tinnitus do exist, a comprehensive review of those guidelines has not been performed. The objective of this review was to identify current clinical guidelines, and compare their recommendations for the assessment and treatment of subjective tinnitus in adults.

**Method:** We systematically searched a range of sources for clinical guidelines (as defined by the Institute of Medicine, United States) for the assessment and/or treatment of subjective tinnitus in adults. No restrictions on language or year of publication were applied to guidelines.

**Results:** Clinical guidelines from Denmark, Germany, Sweden, The Netherlands, and the United States were included in the review. There was a high level of consistency across the guidelines with regard to recommendations for audiometric assessment, physical examination, use of a validated questionnaire(s) to assess tinnitus related distress, and referral to a psychologist when required. Cognitive behavioral treatment for tinnitus related distress, use of hearing aids in instances of hearing loss and recommendations against the use of medicines were consistent across the included guidelines. Differences between the guidelines centered on the use of imaging in assessment procedures and sound therapy as a form of treatment for tinnitus distress respectively.

**Conclusion:** Given the level of commonality across tinnitus guidelines from different countries the development of a European guideline for the assessment and treatment of subjective tinnitus in adults seems feasible. This guideline would have the potential to benefit the large number of clinicians in countries where clinical guidelines do not yet exist, and would support standardization of treatment for patients across Europe.

## Introduction

Tinnitus is essentially made up of two components, the phantom perception of a sound in the ears or head, and the degree of emotional reaction to that percept. Tinnitus can co-occur with several medical-otological disorders such as presbycusis, though etiology is unknown for the majority of tinnitus patients (Baguley et al., 2013b). In rare cases tinnitus indicates a serious underlying pathology such as vascular troubles, vestibular schwannoma (VS), or otosclerosis (Baguley et al., 2013a). In most cases however subjective tinnitus is a benign symptom. In many patients co-morbidities exist such as anxiety, depression, insomnia, and concentration problems, all of which severely impair quality of life (Langguth et al., 2011). In 1–3% of cases tinnitus causes severe health problems, with a wide range of effects on daily life functioning (Davis and Refaie, 2000; Fujii et al., 2011; Kim et al., 2015). Evidence corroborates that the aversive psychological reactions, such as cognitive problems, negative emotions, and dysfunctional attentional processes are of main importance in leading to a severe tinnitus condition (Erlandsson and Hallberg, 2000; Andersson et al., 2006; Cima et al., 2011; Kleinstauber et al., 2013; McKenna et al., 2014; Handscomb et al., 2017).

During the last decades, efforts have been made to better understand tinnitus pathophysiology and provide specialized treatments to patients (Kamalski et al., 2010; Cima et al., 2012; Langguth et al., 2013; Hoekstra et al., 2014). A large number of management strategies including various assessment and treatment procedures exist and have evolved but lack empirical support. For example, there is no evidenced treatment or licensed pharmacological therapy to eliminate the tinnitus percept (Langguth and Elgoyhen, 2012). The Cochrane Library lists 10 completed systematic reviews on different tinnitus treatments, all of which reported small numbers of studies of variable quality (e.g., Martinez-Devesa et al., 2010). These facts combined makes it difficult for healthcare professionals to decide what is best for which tinnitus patient. This is evidenced by the discrepancy between scientific and clinical perspectives on the management of tinnitus and the *actual* day-to-day practice in European healthcare settings (Hoare et al., 2012); tinnitus patient care is fragmented and *ad hoc* (Hoare and Hall, 2011; Hoare et al., 2012). To date there has been no overview of the number of existing clinical practice guidelines for tinnitus, the details included, their comparability, or their purpose. Clinical practice guidelines are defined as systematically developed statements to assist practitioner and patient decisions about appropriate health care for specific clinical circumstances (Field and Lohr, 1990). They have the benefit of simplifying and standardizing assessment and treatment options for clinicians and patients. A European Union guideline would extend this benefit to 28 countries. This systematic review aims to identify, review, and examine the clinical guidelines which do exist for tinnitus. The tinnitus assessments (diagnostics and measures), processes, and treatment options recommended by the respective guidelines will be compared and summarized.

## Methods

The aims, the work plan, and the protocol for this systematic review were developed by TINNET Working Group 1, a COST Action BM1306 (2014–2018) to create a pan-European tinnitus research network (http://tinnet.tinnitusresearch.net/). This review was registered with PROSPERO, the international register of systematic reviews (protocol number: CRD42016038588) prior to commencing the literature search. The review was exempt from human ethics procedures as there were no human participants and only secondary sources of data (the clinical guidelines) were used.

### Eligibility criteria

Records were considered eligible for inclusion if they fit the definition of a guideline by describing and making recommendations on the assessment, diagnosis, and or treatment of subjective tinnitus for adults (i.e., people aged 16 years or older). Those records were required to identify or describe themselves as guidelines, and be the most recent guideline form the country of origin. No publication date or language restrictions were imposed on the eligibility of the guidelines.

Guidelines were excluded if they were for objective tinnitus, pediatrics, referred only to the triage or referral pathways for assessing and treating tinnitus, or if they were a guide for only one specific type of assessment or treatment procedure for tinnitus.

### Literature search

The literature search for clinical guidelines included the Medline, PubMed, and the Cumulative Index to Nursing and Allied Health Literature (CINAHL), and EMBASE databases. In addition to these the National Guideline Clearinghouse (www.guideline.gov), National Institute for Health and Clinical Excellence (NICE; https://www.nice.org.uk/), Guideline International Network (GIN; http://www.g-i-n.net/), Google, and hand-search of reference lists of any included guidelines was undertaken. International experts were also contacted to ask if they were aware of any guidelines that had not already been identified from the search results. The date that the search for guidelines was first conducted was 2 May 2016 and was undertaken by TEF and HH using “tinnitus” and “guideline” as the two key terms. The final search was conducted on 24 June 2016.

### Study selection

Two reviewers independently screened search results by title and abstract, and then by full text if required. The first 20 pages of search results from Google, and all search results from GIN, NICE, and the National Guideline Clearinghouse were screened. In the event of disagreements, a third reviewer (BM) acted as an arbiter. As an additional check and in line with other systemic review searches using internet search engines, a *post-hoc* rule of stopping searching after three consecutive pages without new search results was applied. In this case, no new search results were identified after the first eight pages.

### Data extraction

Data extraction was undertaken using a tailored form that had been pilot tested and was emailed to reviewers in the form of an Excel spreadsheet. A document with guidance on the extraction of information for each of the items was provided to each of the reviewers to improve consistency of data extraction. Data extraction from each guideline was undertaken by at least two reviewers who were native speakers of or fluent in the language in which the guideline was published. Reviewers extracted information from the guidelines regarding items about the: country and year of publication, availability, author details, sponsor/funder involved, scope, target audience, developers and process related to the guideline, recommendations for assessment and treatment procedures, the level of evidence and type of rating system used (e.g., Oxford) related to the recommendations, and items related to the implementation and revision of the guideline.

### Data management

HH and TEF were responsible for data management and maintained editorial rights. All identified records were saved into a Microsoft word master file and then saved in pdf-copy.

### Quality assessment and risk of bias

All reviewers of the guidelines also completed the AGREE II tool (Brouwers et al., 2010) to assess the quality of the guidelines. AGREE II is an international tool to assess the quality and reporting of practice guidelines (www.agreetrust.org). It contains 23 items grouped under six guideline domains. Each item is scored on a 1–7 scale where 1 = “Strongly disagree” and 7 = “Strongly agree.” Scores are standardized to provide an overall percentage score. Previous reviews have used a 60% marker to distinguish high and low quality guidelines (Sanclemente et al., 2014; Ruszczynski et al., 2016).

Details relating to the sources of funding, professional affiliations, and editorial independence of the guideline developers were extracted as indicative of risk of bias.

### Data synthesis

Data extracted by the reviewers were collated and integrated into summary tables and a narrative synthesis describing the similarities and differences between the clinical practice guidelines was completed.

## Results

**Figure 1:**
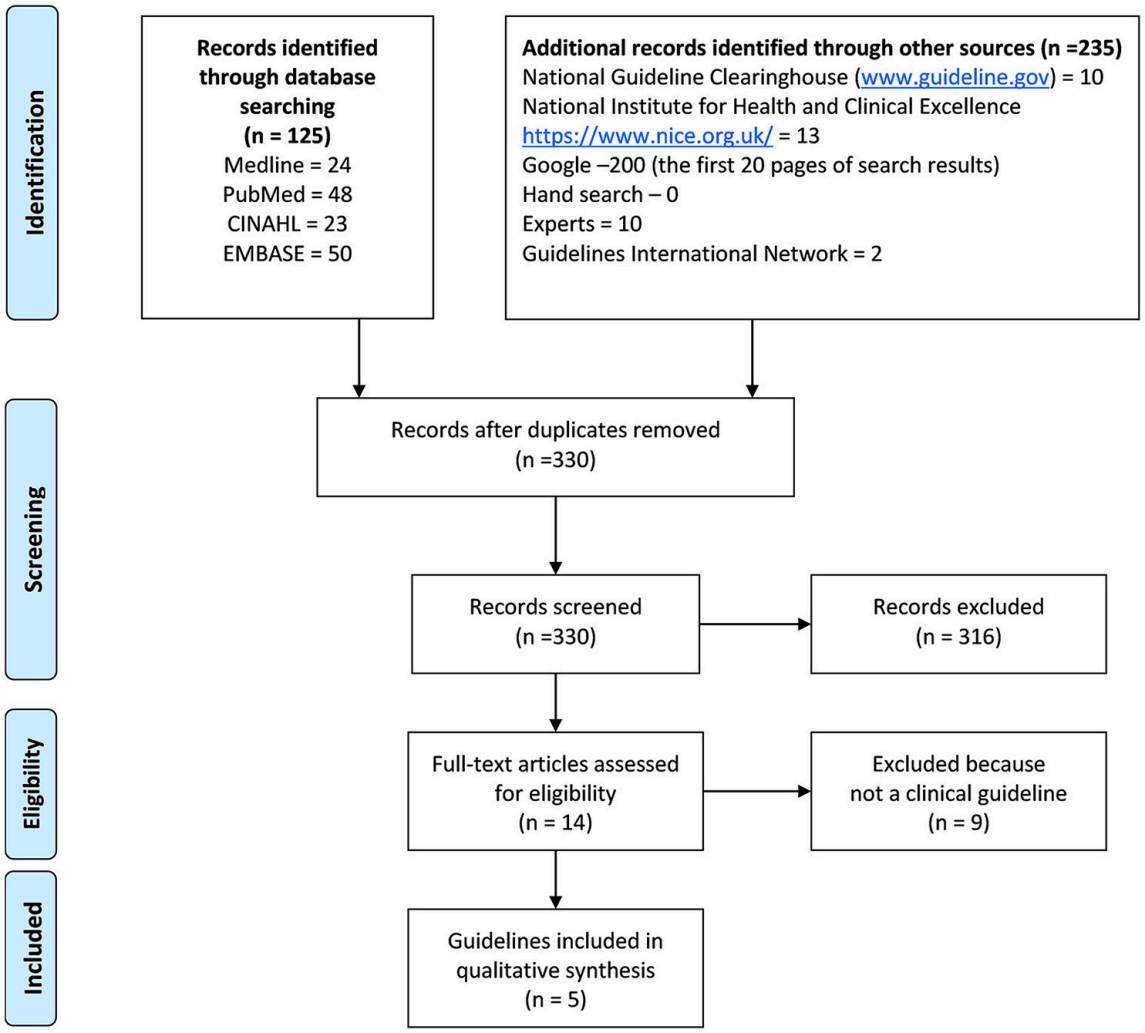
**PRISMA flowchart showing the stages of guideline search, screening and inclusion**.

Five clinical guidelines for tinnitus were ultimately included in this review (see Figure 1 for details of the search and selection process). They were guidelines from Denmark (Jørgensen et al., 2007), Germany (The Association of the Scientific Medical Societies, 2015), The Netherlands (Dutch Association for Ear Nose Throat and Head surgery [Nederlandse Vereniging voor Keel – Neus – Oor heel kunde en Heelkunde van het Hoofd –Halsgebied], in press), Sweden (Idrizbegovic et al., 2011), and United States (Tunkel et al., 2014). Several documents were excluded as by definition not providing a guideline. For example, the Australian audiology clinical practice standards (Audiology Australia, 2013) underwent full-text screening but was not included as it only related to audiological management and had a brief section on tinnitus assessment. The UK Good Practice Guide (Department of Health, 2009) also was excluded as it explicitly states: “This Good Practice Guide to the delivery of services is not, and does not aim to be, an evidence-based guideline for clinical practice with individual patients” (p. 5). The Tinnitus Research Initiative (TRI) algorithm (Biesinger et al., 2010), after some debate within the review team, was also excluded because it was judged not to be a “clinical guideline.” A list of full text documents considered but excluded is in Appendix 1.

Although there was not a restricting time period for the guidelines, no guidelines older than 10 years were identified. With exception of the Danish guideline (published in 2007) all were developed during the last 5 years.

### Details about development of the clinical guidelines

Table 1 provides detailed information about the stakeholder involvement, rigor of development, and the editorial independence associated with the respective clinical guidelines.

Table 1**Summary of guideline development by country**.

**Table:** 

**Country**	**Professionals involved**	**Views of patients considered**	**Target users**	**Views of funding body**	**Competing interests**
Germany	Audiologists, psychiatrist, psychologists, otolaryngologists, dentists, pediatricians, neurologists, and patient representative groups	Patient representative groups were included in the guideline development group; contributed to external review on draft documents, and patient related information was also considered from the results of a literature review	Physicians (especially ENT), phoniatry and pediatric audiology, psychiatry, psychosomatic, neurology, mouth, jaw, and facial surgeons and dentists, psychologists, general practitioners	A statement concerning financial and other interests and editorial independence is included.	Competing interests are declared and when relevant, stakeholders with competing interests were excluded
Denmark	Speech Pathologist and hearing therapists	NS	Hearing therapists	NS	NS
Netherlands	Details provided. ENT-doctors, psychologist, clinical physicist-audiologist	Dutch Association of the Hearing Impaired consulted. A literature review regarding patient preferences was also conducted	ENT doctors, audiology centers, GP's, psychologists, psychiatrists	A statement of independence was signed by professionals involved	Competing interests are declared
USA	Paediatric and adult otolaryngologists, otologists/neurotologists, geriatrician, behavioral neuroscientist, neurologist, audiologist, family physician, radiologist, psychiatrist, psycho-acoustician, nurse, physician, and consumer advocates	Yes: also included a draft of the guideline being made available for public comment	Any clinician, health care provider, specialty physicians, and non-physician providers such as audiologists and mental health professionals	Funded by American Academy of Otolaryngology—Head and Neck Surgery Foundation but no statement of independence from the process	Competing interests are declared
Sweden	Partial details provided—included medical doctors, and professional representatives from the tinnitus teams for diagnostics and rehabilitation	NS	Staff at the audiology and balance clinic at Karolinska University Hospital and professionals that might refer to the clinic (GPs, ENTs or audiologist)	NS	NS

**Table:** 

**Country**	**Methods used**	**Evidence criteria**	Category of evidence^*^	**Strengths and limitations**	**Methods for reaching consensus**	**Consequences of the recommendations**	**Link between evidence and recommendations**	**Peer review?**	**Update of the guidelines?**
Germany	Systematic methods used, details provided in the guideline	Classified according to Oxford Centre of Evidence-based Medicine criteria	1a	Strengths and limitations of the body of evidence are clearly described	Formal consensus technique	The guideline includes health benefits, side effects and risks formulating the recommendations	There is an clear link between the recommendations and the supporting evidence	External review	Due in 2020.
Denmark	Systematic methods used, details provided in the guideline	NS	The guidelines are based on literature, and articles based on the consensus of leading professionals in the field of audiology (evidence level IV)	NS	Informal consensus. All recommendations are based on the ICF model	NS	Each recommendation is provided with an argument based on relevant literature	Not peer reviewed	NS
Netherlands	Systematic methods used, details provided in the guideline	Based on AMSTAR checklist	1a, 1b, IV	The strength of the evidence is specified according to GRADE. Evidence tables describe limitations and strengths of the included studies	Recommendations were evidence based and the importance the workgroup gave to them conforms to GRADE	Recommendations were made considering the scientific value, preferences of the patient, costs, and availability of the organization	There is a clear link between the recommendations and the supporting evidence	External review	Update due in 2020 or sooner if new compelling evidence warrants earlier consideration
USA	Systematic methods used, details provided in the guideline	Based on criteria from the Oxford Centre for Evidence-Based Medicine	American Academy of Pediatrics Categories of evidence (A, B, C, D, and X) updated to be in accordance with Oxford Centre for Evidence-Based Medicine	Strengths and limitations of the body of evidence are clearly described	This guideline was developed using an explicit and transparent a priori protocol for creating actionable statements based on supporting evidence and the associated balance of benefit and harm	The benefits and harms of the recommendations have been considered for each recommendation.	There is an clear link between the recommendations and the supporting evidence	External review	Update due in 2018/9 or sooner if new compelling evidence warrants earlier consideration
Sweden	No method reported	No evidence criteria	No evidence provided	None provided.	NS	NS	NS	NS	NS

* *Unless stated, the level of evidence refers to/uses the Oxford Centre for Evidence Based Medicine criteria (GRADE system consists of 4 grades of degree of trust in conclusions of the literature: high, moderate, low, and very low) NS, not specified; ENT, Ear Nose Throat; GP, General Practitioner*.

All the guidelines included information on the professional backgrounds of the participants in the respective development groups and in three out of the five cases (American, Dutch, and German), provided information on how views of funding bodies and competing interests were addressed. Although patient groups and the public were consulted in the development of three guidelines (American, Dutch, and German), the actual expected users of the guidelines were health professionals.

Details were provided in all guidelines (with the exception of those from Sweden which did not provide methodological information) about how literature was located and used to inform the respective recommendations. That is, details of search strategies using MeSH and other search terms and databases such as Medline and PsychInfo were included. Tools and criteria used to assess the evidence included the: Oxford Centre for Evidence Based Medicine (U.S. and German guidelines) and American Academy of Paediatrics' (American guideline) evidence criteria respectively, the AMSTAR checklist (Dutch guideline), and the GRADE ranking system of trust in conclusions of the literature (Dutch guideline). American, Danish, Dutch and German guidelines all provided information and referred to the research literature associated with each recommendation as well as describing their methods for reaching consensus on each recommendation. The Dutch, German and U.S. guidelines consider the strengths and limitations of the research literature and were reviewed externally prior to publication. Similarly, those three guidelines also state a year by and/or describe conditions under which they would be revised.

#### Assessment recommendations in the clinical guidelines

Table 2 compares assessment recommendations between the respective national tinnitus guidelines. All guidelines, except the Danish, recommend a clinical history (anamnesis/targeted history/special tinnitus anamnesis) be taken.

**Table 2:** **Clinical guideline recommendations regarding assessment of patients with tinnitus**.

**Guideline**	**Physical examination**	**Hearing and audiology tests**	**Psychological assessment**	**Assessment tools/questionnaires recommended**	**Other assessment procedures**	**Procedures not recommended**
Germany	Orientating neurological assessment of cervical spine, vestibular is with examination of denture (including TMJ) in silence to screen modulation of tinnitus Orientating examination of functioning of N. facialis ENT examination including tympanic membrane microscopy, asopharyngoscopy and eustachian respectively stethoscopic examination of the ear and of the carotid artery, particularly in pulsatile tinnitus	Pure tone audiometry discomfort, possibly with categorical loudness scaling determining of tinnitus loudness and frequency using narrow-band noise and pure tones residual inhibition determining the minimum masking level by white noise and pure tones; masking curves according to Feldmann tympanometry and acoustic reflex including recording possible changes due to breathing or heart rate TEOAE and/or DPOAE brainstem auditory evoked response (BAER) preliminary vestibular examination possibly including caloric testing Brainstem audiometry (BERA) when medically justified, economically viable and likely to be useful in informing counseling might be of potential benefit	NS	Goebel-Hiller Tinnitus Questionnaire, VAS or other validated scales	Special tinnitus anamnesis (see Structured Tinnitus Interview (Goebel and Hiller, 2001) X-rays of the cervical spine, if further indicated also functional images	Acoustic examination with more than 84 dB 1 week after acute tinnitus or tinnitus exacerbation
Denmark	NS	Audiometry (performed by ENTs) LDL/UCL If necessary also: ABR	Structured interview	THI-DK VAS-scale for hyperacusis Tværfaglig Tinnitus Screening (Danish tool assessing signs of anxiety)	If necessary also: ABR, CT/MRI, blood samples, other neurological tests	NS
Netherlands	Anamnesis, ENT-assessment inclusive otoscopy and tuning fork tests, Blood pressure measurement, Flexible nasofaryngoscopy, Palpation of neck and area around ear	Audiometry (Air and bone conduction) Speech audiometry	Detailed assessment regarding the nature how tinnitus impacts on daily life and functioning, comorbid symptoms	TQ, mini-TQ THI TFI THQ HADS	MRI/MRA, CT, DSA (angiography)	Not to use MRI with every patient with non-pulsatile, unilateral tinnitus.
USA	Targeted history and physical examination of the head and neck including otoscopy and neurologic examination. When pulsatile tinnitus is reported, the examination should focus on identification of cardiovascular disease and vascular lesions	Prompt, comprehensive audiological examination (Tonal and Speech audiometry and Immittance) in patients with tinnitus that is unilateral, persistent (≥6 months), or associated with hearing difficulties (Strong recommendation); Initial comprehensive audiological examination (including ear specific masked air and bone conduction) in patients who present with tinnitus regardless of laterality, duration, or perceived hearing status (Option)	Distinction between patients with bothersome tinnitus from patients with non-bothersome tinnitus. Assess degree of tinnitus related disability (including baseline measurement for the purpose to establish effects of treatment). Assess if further psychological treatment required	TQ, TEQ, THQ, TRQ, THI, TFI	NS	Imaging studies unless patients have one or more of the following: tinnitus that localises to one ear, pulsatile tinnitus, focal neurological abnormalities, or asymmetric hearing loss
Sweden	NS	Audiometry (including LDL when necessary) Speech and speech in noise test and impedance audiometry ABR and MRI when necessary	In case of severe tinnitus: the first encounter with the psychologist/ psychiatrist is investigative and informative. (1) symptoms tinnitus, (2) individual's mental status, (3) the overall life situation	BAS (basic own questionnaire), THI HADS (when necessary)	Anamnesis focused on tinnitus onset, laterality, character and patients' problems. Consideration of psychological factors and somatosensory factors	NS

*ABR, Auditory brainstem response; BAER, Brainstem auditory evoked response; CT, Computer tomography; DSA, Digital subtraction angiography; DPOAE, Distortion product optoacoustic emission; ENT, Ear nose throat; GP, General Practitioner; HADS, Hospital Anxiety and Depression Scale; LDL, Loudness discomfort level; MRA, Magnetic resonance angiography; MRI, Magnetic resonance imaging; TEOAE, Transient evoked optoacoustic emission; TEQ, Tinnitus evaluation questionnaire; TFI, Tinnitus functional index; THI, Tinnitus handicap inventory; THQ, Tinnitus handicap questionnaire; TMJ, Temporomandibular joint; TQ, Tinnitus questionnaire; TRQ, Tinnitus reaction questionnaire; UCL, Uncomfortable listening level; VAS, Visual analog scale*.

All guidelines describe the need for physical examination by an ENT doctor, although physical examination is not explicitly referred to in the Swedish guideline. The American guideline recommends examination to exclude objective tinnitus, cardiovascular disease and vascular lesions, neurologic diseases, middle or outer ear infection/disease, vertigo, head-neck masses, or other treatable conditions. The German guideline additionally mentions cervical, dental, and temporomandibular joint functional exploration in a silent environment to evaluate tinnitus modulation.

Audiological assessment was recommended in all the included guidelines. The majority refers to audiometry as a general category, but the German guideline provides most detail. For example, it specifies details relating to the assessment of oto-acoustic measurements, brainstem auditory evoked responses, caloric tests, determination of tinnitus loudness and frequency using narrow-band noise and pure tones, residual inhibition, Feldmann masking curves (Feldmann, 1984), and loudness discomfort level. None of the other guidelines included in this review recommend psychoacoustic measurements of tinnitus frequency or intensity.

The German guideline does not refer to specific psychological assessments though the other guidelines do in varying terms. For example, when tinnitus is severe or accompanied by psychological factors, the Swedish guideline recommends psychological assessment while the Danish guideline recommends a structured interview. The American guideline on the other hand recommends that clinicians distinguish between patients with or without bothersome tinnitus for subsequent referral (when necessary) to a psychologist or psychiatrist.

The Tinnitus Handicap Inventory (THI; Newman et al., 1996, 1998) is the most frequently referred to assessment questionnaire followed by Tinnitus Questionnaire (TQ; Goebel and Hiller, 1994). Visual Analog Scales (VAS; e.g., Germany, Denmark) and the Hospital Anxiety and Depression Scale (HADS; Zigmond and Snaith, 1983, e.g., The Netherlands, Sweden) were referred to by at least two guidelines. The American guideline referred to a large number of questionnaires including the: TQ (Goebel and Hiller, 1994), THI (Newman et al., 1996, 1998), Tinnitus Effects Questionnaire (TEQ; Hallam et al., 1988), Tinnitus Handicap Questionnaire (THQ; Kuk et al., 1990), Tinnitus Reaction Questionnaire (TRQ; Wilson et al., 1991), and Tinnitus Functional Index (TFI; Meikle et al., 2012).

Several guidelines make recommendations for or against the use of other assessment related procedures. For example, the German guideline refers to X-rays of the cervical spine. Although three guidelines recommend magnetic resonance imaging (MRI) as an assessment of tinnitus, The American and the Dutch guideline recommend against it, unless patients have one or more of: tinnitus that localizes to one ear, pulsatile tinnitus, focal neurological abnormalities, or asymmetric hearing loss. The German guideline also recommends against acoustic examination using sound pressure levels more than 84 dB 1 week after acute tinnitus or tinnitus exacerbation.

#### Summary of recommendations regarding the assessment of subjective tinnitus

- Conduct a thorough physical examination to exclude possible (physical) causes of tinnitus (three of five guidelines; not stated in Danish and Swedish).
- Complete a thorough audiological assessment (all guidelines).
- Establish the degree to which a patient experiences subjective tinnitus as bothersome or distressing using a validated and reliable multi-item questionnaire such as the TQ, THI, TFI, or HADS (all guidelines).
- In situations where patients appear to be experiencing a degree of distress or difficulties related to living with tinnitus, consider making a referral for an assessment by a psychologist or psychiatrist (four of five guidelines; not stated in German guideline).
- Variation exist in recommendations regarding the use of imaging studies (e.g., MRI).

#### Treatment recommendations across the guidelines

Table 3 compares therapeutic recommendations for the treatment of subjective tinnitus between the respective national tinnitus guidelines; note the Danish guideline is not included in this table as it provides only recommendations regarding assessment procedures. Across the guidelines there is generally a high degree of consistency in the recommendations for or against: the use of medicines (prescribed drugs and herbal supplements); audiological and psychological interventions; and, transcranial magnetic stimulation. Greatest variation occurs in the recommendations concerning the use of therapies involving sound such as Tinnitus Retraining Therapy (TRT).

**Table 3:** **Tinnitus guideline recommendations regarding treatments for tinnitus**.

**Country**	**Medicine**	**Audiological**	**Psychological**	**Sound therapies**	**Other**	**Treatments recommended against using**
Germany	None for tinnitus but refers to, for example, some for co-morbid depression, e.g., glutamate-antagonists.	Hearing aids for patients with hearing loss; Cochlear Implants for patients with deafness.	General counseling (including information provision). Tinnitus specific CBT (aimed at reducing attention focusing toward the ear noise, reappraisal of the tinnitus and its consequences) individual or group-settings, also treatment for comorbidities. Hospital treatment for decompensated tinnitus and/or with severe psychiatric comorbidity. An absence of conclusive evidence of effectiveness for self-help groups.	Audio therapy including “notched music,” “coordinated reset” or music therapy.	Absence of evidence of effectiveness for: acupuncture, cervical vertebral spine therapy/physiotherapy, hyperbaric oxygen; and, electric stimulation (e.g., transcutaneous electric stimulation, ear and cervical spine; vagus stimulation); Acoustic Coordinated-Reset Neuromodulation. Uncertain recommendation for rTMS.	Sound therapy including Noiser and TRT. Hearing aids for patients with only tinnitus. Medicines (including: steroids, melatonin, antidepressants, Sulpirid, Apraxolam, Sertraline, Botox A, Pramiprexol, Nortriptyline, Piribedil, Vardenafil, Trazodone, Atorvastatin, Gabapentin, anticonvulsants, Paroxetine, Lamotrigine, Cyclandelat, Baclofen, Nicotinamide, Tocainid, Misoprostol, Egb 761, Amitriptyline, Misoprostol, Pramipexole Dopamine. Herbal medicines and vitamins (including Ginkgo biloba zinc).
Netherlands	None	Consider a trial of hearing aids. In patients with high TQ (>60) or THI (>78) scores, and have severe hearing loss or deafness and have not responded to CBT, consider Cochlear Implant.	Educational material about tinnitus and treatment options considered essential. Specialised CBT for patients with TQ > 30 or THI > 36.	TRT can only be contemplated in case tinnitus is very mild (TQ <30) and the patients specifically asks for TRT.	None	rTMS. TDCS. Gingko biloba. Acupuncture. Auditive perceptual training. Hyperbaric oxygen.
Sweden	None for tinnitus specifically but does state that if necessary, sleeping pills or antidepressants, can be used to treat sleep disorders or depression (no drug types, names, or dosage provided).	For people with tinnitus and hearing loss hearing aids are fitted.	Individual or group tinnitus information meetings. For patients without hearing loss, this is based on a modified version of TRT protocol. There is reference to CBT in case of stress/anxiety/depression, but no clear recommendation.	Sound stimulation as part of TRT for people without hearing loss.	For middle ear dysfunctions such as otosclerosis, surgery is possible – no clear recommendation is provided. For tensions or pain in the jaw, neck, shoulders or back, referral to “bite” therapist, or physiotherapist.	None
USA	None	Clinicians *should* recommend a hearing aid evaluation for patients with hearing loss and persistent, bothersome tinnitus.	Clinicians *should* educate patients with persistent, bothersome tinnitus about management strategies. Clinicians *should* recommend cognitive behavior therapy to patients with persistent, bothersome tinnitus.	Clinicians *may* recommend sound therapy (e.g., TMT, TRT) to patients with persistent, bothersome tinnitus but patients must be informed of potential outcomes as well as costs associated with sound therapy.	*No recommendation* can be made regarding the effect of acupuncture in patients with persistent bothersome tinnitus based on the poor quality of trials, no benefit, and minimal harm.	Clinicians *should not* routinely recommend: Medicine (including antidepressants, anticonvulsants, anxiolytics, or intratympanic medications for a primary indication of treating persistent, bothersome tinnitus. Dietary supplements and herbal medicines (e.g., Ginkgo biloba, melatonin, zinc). TMS.

*CBT, Cognitive behavioral therapy; NS, not specified; rTMS, repetitive Transcranial magnetic stimulation; TCDS, Transcranial direct stimulation; THI, Tinnitus handicap inventory; TMS, Transcranial magnetic stimulation; TMT, Tinnitus management therapy; TQ, Tinnitus questionnaire; TRT, Tinnitus retraining therapy*.

There is a consensus that medicines should not be prescribed for the treatment of subjective tinnitus, though some variation in the level of specificity that each guideline has. For example, the German guideline lists specific medicines that should not be prescribed for the treatment of tinnitus. The German and Swedish do however note that medicines such as antidepressants might be prescribed to treat comorbid conditions. Herbal supplements such as Gingko biloba are also specifically recommended against being used in all guidelines except for Sweden which does not make recommendations for or against their use.

The use of hearing aids is recommended by all guidelines but only when clinically meaningful hearing loss is also present in people suffering from tinnitus. The use of a cochlear implant is mentioned in the Dutch and German guidelines and only recommended when there is profound hearing loss or deafness in addition to tinnitus. The Dutch guideline is the only one to provide scores on tinnitus questionnaires (e.g., TQ, THI) for when such interventions should be considered (e.g., it recommends referral to specialized stepped-care CBT for tinnitus in cases where TQ score is greater than 30, in combination with a clinically relevant request for healthcare by the patient, as is judged by the referring party).

Psychological interventions for tinnitus can potentially include a wide range of components but there is general consensus on the use of two of them. In particular, the provision of information and education about tinnitus and treatment options is consistently recommended across the guidelines although there is some variation in the specificity of the content that each provides. Second, specialized CBT for tinnitus is specifically recommended by all the guidelines except for Sweden which mentions it only in relation to the presence of stress, anxiety, or depression.

Least consistency exists across the guidelines in relation to TRT. Specifically, the Dutch guideline recommends that TRT can only be contemplated if tinnitus is very mild (TQ <30) and the patients specifically asks for TRT, the American guideline indicates that sound therapies “may” be recommended to patients with tinnitus, while the Swedish guideline recommends that sound stimulation be used as part of TRT for people without hearing loss. The German guideline recommends the use of notched music therapy, but recommends against the use of TRT.

In relation to other less commonly used treatments (such as acupuncture or hyperbaric oxygen), the guidelines mostly indicate that there is an insufficient body of evidence to be able to make recommendations for or against their use.

Lastly, three guidelines (Germany, The Netherlands, and U.S.) either caution that there is insufficient evidence, or make additional recommendations against the use of transcranial magnetic stimulation (rTMS), transcranial direct current stimulation (tDCS), dietary supplements, neuromodulation treatments, and hearing aids for tinnitus patients without hearing loss.

#### Summary of therapeutic recommendations regarding the treatment of subjective tinnitus

- Provide information about tinnitus and treatment options (all guidelines).
- Use hearing aids only when patients also experience hearing loss (all guidelines).
- Specialised CBT for tinnitus should be offered to patients (three of four guidelines; Sweden refers to use of CBT in context of co-morbid anxiety or depression).
- There is a lack of consensus on the use of TRT for tinnitus.
- Prescribed medicines and herbal supplements should not be used for the treatment of tinnitus (all guidelines).
- Treatment with TMS is recommended against by Dutch and U.S. guidelines, and German guidelines give an “uncertain” recommendation.

#### Quality assessment of the guidelines

The AGREE II tool was used by the authors who undertook data extraction of the respective guidelines and the summarized results are shown in Table 4. In general the domains of “stakeholder involvement” and “clarity of presentation” respectively by guideline developers were rated high (good quality). Conversely, ratings on the domain of “applicability” which refers to how the guidelines might be disseminated, implemented and evaluated were low. For the domains addressing the scope and purpose of the guidelines, rigor of development and editorial independence, a pattern emerged whereby the American, Dutch and German guidelines were rated considerably higher (AGREE II scores >60% on all domains) than the Danish and Swedish guidelines (AGREE II scores <60% on all domains).

**Table 4:** **Summary of AGREE II domain scores (%) by country**.

	**Scope & Purpose**	**Stakeholder involvement**	**Rigour of development**	**Clarity of presentation**	**Applicability**	**Editorial independence**
Germany	61	94	83	89	71	67
Denmark	52	44	24	59	2	17
Netherlands	81	100	97	100	9	100
USA	86	97	93	100	71	88
Sweden	42	42	1	33	2	13
Median	61	94	83	89	9	67
Average	64	75	60	76	31	57

## Discussion

This systematic review aims to compare existing clinical guidelines for the assessment and treatment of subjective tinnitus in adults. Five guidelines, developed in the last 10 years within Europe, Scandinavia, and North America were included in the review. Although there are differences in some specific recommendations for assessment and treatment procedures across the guidelines, in general, commonalities across guidelines were high. The fact that there are differences in some of the recommendations is not surprising and appears to reflect the relatively young state of the field and the evolving nature of assessment and treatments for subjective tinnitus—a symptom with a high level of heterogeneity. On the other hand, the level of agreement, for example, in the recommendation of specialized cognitive behavioral therapy reflects the growing evidence base for the effectiveness of this treatment to alleviate patients' distress and impairment, even though significant changes in the tinnitus percept itself as a result of CBT have been proposed, though not yet assessed across studies.

When the methods of the development of guidelines were reported, it was clear that the respective groups were making efforts to be transparent, systematic, and using the best available evidence base, and frequently linking recommendations to specific research literature. For example, systematic reviews and meta-analyses were referred to whenever available to inform recommendations. It should be noted though that there is a lack of high quality studies or powered randomized trials of some treatments either for practical or methodological reasons. Regardless, the strengths and limitations of the evidence for particular recommendations were included for the majority of the guidelines and thus enable the user/reader to make informed decisions about following the recommended actions. Furthermore, target users were generally clearly defined and the development groups were comprised of a range of the health professionals often involved in the assessment and treatment of tinnitus. These two factors are important not only for providing expert input into the guideline, but also for garnering “buy-in” from potential users of the guidelines and focussing the content.

### Differences between the guidelines

Differences in recommended assessment procedures tend to relate to specific techniques (questionnaires, diagnostic tests, types of scanning techniques) rather than general principles [e.g., trying to establish tinnitus severity, hearing loss, psycho-social problem(s)], or the presence or absence of severe physical pathology that might be causing the tinnitus. Differences related to, for example, the recommended questionnaires for assessing tinnitus related interference and distress. While all the guidelines referred to the THI (the German guideline indirectly refers to this), only the American, Dutch and German guidelines referred to the TQ. Recommendations for specific questionnaires to measure psychological distress (especially symptoms of anxiety and depression) also varied with some guidelines not mentioning any (e.g., United States) and others such as the Dutch and Swedish guidelines which referred to the HADS. Differences also existed between the recommendations to assess loudness discomfort levels with the American and Dutch guidelines not recommending the use of such tests while the other guidelines did.

With regard to treatments, differences are found primarily regarding recommendations for the use of sound therapies. TRT specifically is not recommended by the German guideline, conditionally by the Dutch guideline and the American guideline indicates that clinicians “may” recommend it; TRT is currently being tested in a large multicenter trial in the U.S. (clinical trials ID: NCT01177137). A lack of evidences about other treatments such as acupuncture, hyperbaric oxygen and some herbal supplements leads most groups to recommend against them. The American guideline though is more cautious and simply states that because there is a lack of evidence they can neither recommend for or against the use of such treatments.

Differences in the recommendations of assessment and treatment procedures could be explained by a combination of factors including the time of the development of the guideline and availability of translated versions of the questionnaires (e.g., the TFI was published in 2012 which was after that of the Danish and Swedish guidelines), the known psychometric properties of the questionnaires themselves [e.g., concerns have been raised about the cross-cultural use of the HADS (Maters et al., 2013)], and the different methods used to reach consensus by the different guideline groups.

### Consistencies across the guidelines

Across the guidelines consensus appears to exist on a number of important general features of assessment relating to subjective tinnitus. Specifically, there is consensus about the initial need for excluding a physical cause of the tinnitus, conducting an audiometric assessment of the patient, using standardized questionnaires to measure degrees of tinnitus related distress, and when relevant, making referrals for further psychological assessment.

Regarding the therapeutic recommendations for the treatment of subjective tinnitus, all guidelines recommend against the use of medicines for the treatment of the tinnitus specifically but note that medicines are appropriate for treating co-morbid conditions. There is also agreement in the recommendations to use hearing aids for patients experiencing hearing loss and CBT to facilitate adjustment to the symptom, alleviate distress and tinnitus-related interference in daily life.

As a group of tinnitus researchers and clinicians, we endorse the specific principles and practices of assessment and treatment that are consistently found across the guidelines. Further, while a treatment for removing the tinnitus percept does not exist, we reiterate the importance of providing patients with bothersome tinnitus, evidence based cost-effective treatment(s) in a way (such as stepped care) that is minimally burdensome to the patient. That is patients who are assessed as having relatively little tinnitus related distress and interference should receive less intensive treatment in the first instance, than someone who is assessed as having severe levels of distress and interference in activities of daily living.

### Strengths and limitations of the review

There are two critical factors that affect the conclusions that can be drawn from the included guidelines. Firstly, and as with all systematic reviews, the search strategy and inclusion criteria used determine what is located and subsequently included. In this review, we used the search terms “tinnitus” and “guideline” to conduct the search in a wide range of databases, repositories of clinical guidelines, and search engines, with the intention of being focussed enough to identify the most relevant documents within a manageable number of search results. Only including the term “guideline” though might have resulted in relevant documents, albeit not called “guidelines,” being omitted from search results. Similarly, our use of inclusion/exclusion criteria that led to the decision to exclude documents such as the TRI flowchart could be problematic as it (the TRI flowchart) is a comprehensive document potentially used in many situations to inform assessment and treatment decisions.

To minimize the risk of omitting relevant search results we contacted a range of international experts and members of guideline development groups. In addition to this, we conducted hand searches of the references lists of included guidelines for relevant sources. We also recruited native speakers to extract data from the respective guidelines in an effort to ensure that data collection was as accurate as possible. It is possible, that different search and inclusion criteria might have led to different documents being included. However, given the large range of assessment and treatment options and the limited evidence base around many treatments in particular, it is unlikely that our conclusions would differ significantly if further guidelines had been identified at this time. Future systematic reviews though will be able to use this as a reference point.

## Implications and conclusions

As researchers from around the world are collecting and making efforts to better understand the heterogeneity of subjective tinnitus in adults and systematically evaluate assessment and treatment options, we have, for the first time, described the major similarities and differences between existing clinical guidelines for subjective tinnitus in adults. The results reveal true guidelines from only five countries and thus highlight a need to develop guidelines that are endorsed by the range of professionals involved in assessing and treating tinnitus. Although we do not place a great deal of weight on the quality assessment ratings of the guidelines, they do suggest that there is room for improvement particularly with regard to implementation and evaluation. The absence of guidelines contributes to the variations that exist in assessment and treatment of tinnitus internationally.

While it would be tempting to do so, it is beyond the scope of this paper to formulate a new or composite guideline based on the results that we have obtained. Instead, the results of this review in conjunction with those from a survey of European tinnitus healthcare providers and researchers (Cima et al., 2016) will form the basis of further work on the development of a set of European clinical guidelines for the assessment and treatment of tinnitus being undertaken by the COST-action TINNET: Working Group I “*Clinical*.” As with existing clinical guidelines, attention will need to be given to how the future European guideline is disseminated, subsequently evaluated, and the implications for resource management considered. We expect it to be challenging task but one that will hopefully result in a more reliable and equitable assessment and treatment of tinnitus patients across Europe.

## Author contributions

TF, HH, DK, AL, BM, AN, SR and RC, conceived and designed the study. TF and HH wrote and revised the protocol and manuscripts, conducted the literature search, data extraction, and data synthesis. SR, RL, CC, NE, PB, SNR, AK, DL, and BM extracted data. DH, AN, DK, and RC reviewed and edited manuscripts and contributed intellectually to the content of the manuscript. All authors approved the manuscript for submission.

## Funding

This research was supported by funding from SWOL Limburgs Fonds voor Revalidatie and Adelante, Centre for expertise in Rehabilitation and Audiology. A COST Action grant (BM1306) supported the collaboration between the authors and the formation of the COST Action BM1306 (2014–2018) TINNET Working Group I.

### Conflict of interest statement

The authors declare that the research was conducted in the absence of any commercial or financial relationships that could be construed as a potential conflict of interest.

## Appendix

Excluded full text documents

1. UK Good practice guide for adults with tinnitus (Department of Health, 2009).
2. Audiology Australia Professional Practice Standards - Part B Clinical Standards (Audiology Australia, 2013).
3. American Speech language and hearing association Tinnitus triage guidelines (American Speach Language Hearing Association, 2016).
4. Ear care, NHS Scotland General practice guide for ear care. (NHS Scotland, 2006).
5. TRI flowchart (Biesinger et al., 2010).
6. Clinical guide for audiologic tinnitus management: Assessment and Clinical guide for audiologic tinnitus management: Assessment (Henry et al., 2005a) and Treatment (Henry et al., 2005b).
7. Adult Tinnitus Management Clinical Practice Recommendation (Henry et al., 2015).
8. American Academy of Audiology Audiologic Guidelines for the Diagnosis and Management of Tinnitus Patients (American Academy of Audiology, 2000).
